# Cordycepin mediates neuroprotection against apoptosis via ERK/CREB signaling activation in Aβ_1–42_‐induced neuronal cell models

**DOI:** 10.1002/ibra.12192

**Published:** 2025-02-08

**Authors:** Wenshu Zhou, Cheng Wang, Yige Tan, Philip Lazarovici, Xiaoyan Wen, Shaoping Li, Wenhua Zheng

**Affiliations:** ^1^ Faculty of Health Sciences, and Zhuhai UM Science & Technology Research Institute University of Macau Macau SAR China; ^2^ State Key Laboratory of Primate Biomedical Research, Institute of Primate Translational Medicine Kunming University of Science and Technology Kunming China; ^3^ State Key Laboratory for Quality Research in Chinese Medicine University of Macau Macao SAR China; ^4^ School of Pharmacy Institute for Drug Research, Faculty of Medicine The Hebrew University of Jerusalem Jerusalem Israel; ^5^ Zhongshan Key Laboratory of Zebrafish‐based Drug Innovation, ZebraPeutics (Zhongshan) Ltd. Zhongshan China

**Keywords:** Aβ_1–42_‐induced apoptosis, cordycepin, ERK/CREB pathway, neuronal cell lines, neuroprotection

## Abstract

The aggregation of β‐amyloid (Aβ) peptides has been associated with the onset of Alzheimer's disease (AD) by causing neurotoxicity due to oxidative stress and apoptosis. Cordycepin is a natural derivative of the nucleoside adenosine that displays potent antioxidant, antitumor, anti‐inflammatory, and neuroprotective properties. However, the mechanism of the neuroprotective effect of cordycepin toward Aβ‐induced neurotoxicity, as well as underlying mechanisms, is still unclear. In this study, we found that cordycepin conferred neuroprotection to catecholaminergic PC12 neuronal cell cultures exposed to Aβ_1–42_‐insult by reducing the production of reactive oxygen species, restoring the mitochondrial membrane potential, and inhibiting apoptosis. Cordycepin stimulated the phosphorylation of extracellular signal‐regulated kinase (ERK) and cyclic AMP‐responsive element‐binding protein (CREB) in a time‐ and concentration‐dependent manner. Inhibition of the ERK pathway reduced the neuroprotective effect of cordycepin. Similar results were obtained with hippocampal HT22 neuronal cell cultures. Cumulatively, these findings suggest that cordycepin‐induced neuroprotection toward Aβ_1–42_ neurotoxic insult may involve activation of the ERK/CREB pathway. This study expands our knowledge of the neuroprotective function of cordycepin and suggests that it holds promise as a natural lead compound for drug development in AD.

## Graphical abstract

This study demonstrates cordycepin's neuroprotective effects against Aβ_1–42_‐induced apoptosis in neuronal cells, mediated through the activation of the extracellular signal‐regulated kinase/cyclic AMP‐responsive element‐binding protein (ERK/CREB) signaling pathway. These findings highlight cordycepin's potential as a therapeutic agent for Alzheimer's disease and other neurodegenerative disorders, warranting further investigation into its mechanisms and applications.

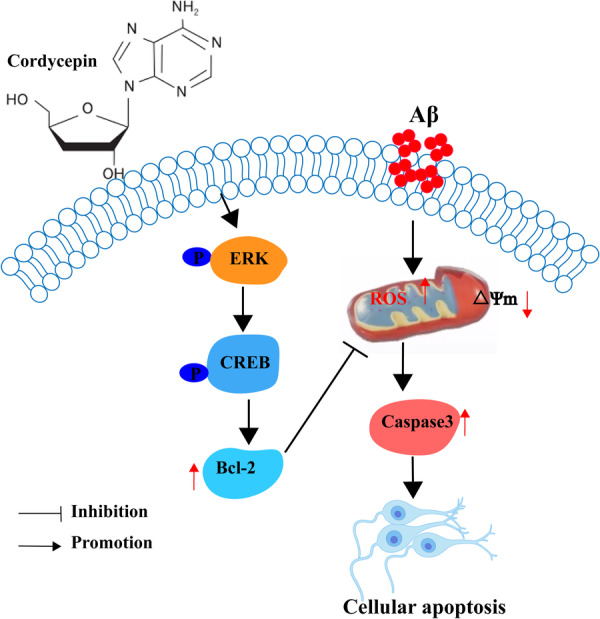

## 1 INTRODUCTION

Alzheimer's disease (AD) is a complex brain disease with progressive and irreversible neuronal cell death,^1^ associated with abnormalities in the Tau and β‐amyloid (Aβ) proteins, which lead to their deposition as Aβ plaques and neurofibrillary tangles. Tau and Aβ proteins are considered to be one of the caspase activators,^2^ and therefore, apoptosis caused by Aβ proteins is an important cause of neuronal cell death.^3^ Aβ is a peptide composed of 39 to 43 amino acids, which accumulates in large amounts and forms deposits in the brain tissue of patients with AD.^4^ In the brain, Aβ primarily exists in two forms: Aβ_1–40_ and Aβ_1–42_, among which Aβ_1–42_ contains all amino acid sequences that are the main constituent of Aβ plaques and imitate the action of Aβ.^4^, ^5^, ^6^ Aβ‐induced neurotoxicity involves damage to mitochondrial function resulting in apoptosis of cells^7^ and neuronal loss,^8^ increased reactive oxygen species (ROS), shrinkage of the nucleus, and formation of cellular debris. Due to the variety of ways cell death affects the development of AD, chemicals inhibiting apoptosis could potentially be useful in the treatment of this condition. Therefore, there is a constant need to search for novel apoptosis inhibitors and evaluate them in preclinical studies and clinical trials.

Cordycepin is an adenosine analog (3′‐deoxyadenosine) extracted from the Cordyceps militaris^9^ that has been used in traditional Chinese medicine and is characterized by multiple pharmacological actions such as antitumor,^10^, ^11^ anti‐inflammatory,^12^, ^13^ antioxidant,^14^ and antiaging activities.^15^ Different studies indicated that cordycepin has neuroprotective effects in a rotenone‐induced Parkinson rat model via an antiapoptotic mechanism^16^ and protected synaptic function and dendrite morphology from cerebral ischemia injury in a rat model of common carotid arteries occlusion.^17^ Several have studies indicated that cordycepin reduces Aβ precursor protein synthesis in human neuroblastoma SH‐SY5Y cell cultures,^18^ delayes Aβ_25–35_‐induced neuronal membrane depolarization,^19^ and mitigated the oxidative stress and the abnormal calcium homeostasis‐dependent cytotoxicity in the hippocampal CA1 pyramidal neurons.^20^ However, its multitarget neuroprotective mechanisms of action remain largely unknown.

Mitochondria in neurons play an important role in the regulation of neuronal metabolism, regulation of synaptic signal transmission, neuronal plasticity, and regulation of ROS levels.^21^ Mitochondrial dysfunction is a central pathology of AD, stemming from various factors such as mitochondrial DNA damage, oxidative stress from reactive oxygen and nitrogen species, membrane and ionic gradient destabilization, and interaction with toxic proteins such as Aβ,^22^ triggering neuronal apoptosis.^23^ Considering that cordycepin exhibited a significant antioxidant and antiapoptotic potential in many diseases, it would be important to know whether cordycepin can offer protection to brain neurons from oxidative stress and mitochondrial damage induced by Aβ.

It is widely acknowledged that the activation of extracellular signal‐regulated kinase (ERK) by phosphorylation stimulates its downstream target, Ser133‐phosphorylated cyclic AMP‐responsive element‐binding protein (CREB),^24^ providing protection against oxidative stress and mitochondrial‐dependent apoptosis.^25^ Activation of ERK‐CREB signaling enhances neuronal survival, inhibited apoptosis,^26^, ^27^ and promoted neuronal differentiation as expressed by neurite outgrowth,^28^ indicating its importance as a crucial signaling pathway for antioxidant and antiapoptotic effects.

To further extend the knowledge of cordycepin neuroprotective effect and its mechanisms of action toward Aβ_1–42_‐induced neurotoxicity, in the present study, we investigated in vitro the AD models of sympathetic PC12^29^ and hippocampal HT22 cell line cultures.^30^ In these cultures, exposed to Aβ_1–42_ insult, cordycepin induced a significant decrease in ROS levels and cell apoptosis and restored the mitochondrial membrane potential. Moreover, cordycepin stimulated the phosphorylation of ERK and CREB, and inhibition of the ERK pathway reduced its neuroprotective effect. These findings support the hypothesis that cordycepin‐induced neuroprotection toward Aβ_1–42_ neurotoxicity may involve activation of the ERK/CREB signaling pathway.

## 2 MATERIALS AND METHODS

### 2.1 Materials

Aβ_1–42_ was purchased from ONTORES Biotechnologies Company and detailed information is shown in Table 1.^31^ Dimethyl sulfoxide (DMSO), Dulbecco's modified Eagle's medium (DMEM), poly‐d‐lysine, 3‐(4,5‐dimethylthiazol‐2‐yl)‐2,5‐diphenyltetrazolium bromide (MTT), CellROX® Deep green Reagent (ROS), and 5,5′,6,6′‐tetrachloro‐1,1′,3,3′‐tetraethyl‐benzimidazolyl‐carbocyanineiodide (JC‐1) were obtained from Byotime, China. The fluorescently labeled annexin V/propidium iodide (Annexin V‐FITC/PI) Apoptosis Detection Kit was sourced from BD Biosciences. Fetal bovine serum (FBS) and 0.25% Trypsin were acquired from Life Technologies. PD98059 was obtained from Merck Millipore. Anti‐p‐ERK1/2, anti‐ERK1/2, anti‐p‐CREB (Ser133), anti‐cleaved caspase‐3, and anti‐glyceraldehyde‐3‐phosphate dehydrogenase (GAPDH) were purchased from Cell Signaling Technology. The Anti‐Rabbit IgG HRP‐conjugated secondary antibody was procured from Promega.

**Table 1:** Detail information of β‐Amyloid_1–42_.

Sequence (three‐letter code)	H‐Asp‐Ala‐Glu‐Phe‐Arg‐His‐Asp‐Ser‐Gly‐Tyr‐Glu‐Val‐His‐His‐Gln‐Lys‐Leu‐Val‐Phe‐Phe‐Ala‐Glu‐Asp‐Val‐Gly‐Ser‐Asn‐Lys‐Gly‐Ala‐Ile‐Ile‐Gly‐Leu‐Met‐Val‐Gly‐Gly‐Val‐Val‐Ile‐Ala‐OH
One letter code	DAEFRHDSGYEVHHQKLVFFAEDVGSNKGAIIGLMVGGVVIA
Molecular formula	C_203_H_311_N_55_O_60_S
Molecular mass	4514.10

### 2.2 Methods

#### 2.2.1 Cell cultures

PC12 cells, derived from a transplantable rat pheochromocytoma, were obtained from Yat‐sen University's Cell Bank. PC12 cells were cultured in 75‐cm^2^ flasks in DMEM supplemented with 5% FBS, 5% horse serum, 100 μg/mL streptomycin, and 100 U/mL penicillin and incubated at 37°C with 5% CO_2_ humidified atmosphere. The medium was replaced every 2–3 days. Cells were applied to 96‐well, 48‐well, six‐well plates or 10 cm dishes coated with 10 µg/mL poly‐d‐lysine and allowed to grow for at least 24 h.

HT22 cells, an immortalized mouse hippocampal neuronal cell line commonly used as an in vitro model for neurodegenerative diseases,^32^ were obtained from Yat‐sen University's Cell Bank (Guangzhou, China). HT22 cells were cultured in 10 cm plates with DMEM containing 10% FBS and 0.1% penicillin/streptomycin in an incubator (Thermo Fisher Scientific) set at 37°C with a 5% CO_2_ humidified atmosphere. The culture medium was refreshed every 3 days, and cell density was maintained at 80%–90%. Passage was performed using 0.25% trypsin when the cells reached the desired confluence. After trypsin digestion, cells were collected by centrifugation at 1000 rpm for 3 min and then resuspended in a fresh medium. Subsequently, the cells were applied to new plates for further experiments.

#### 2.2.2 Cordycepin solution

Cordycepin was obtained from the Institute of Chinese Medical Sciences at the University of Macau (Macau, China), and a 100 mM cordycepin storage solution was prepared using DMSO (67‐68‐5, Sigma‐Aldrich) for the upcoming experiments.

#### 2.2.3 MTT assay

The MTT assay is commonly used to assess cell viability.^33^ The cell cultures, at a concentration of 5 × 10³, were applied to 96‐well plates containing 10% FBS for 24 h. Subsequently, the cells were treated with or without different doses of Aβ_1–42_ for an additional 24 h. In another set of experiments, the cell cultures were treated with or without different concentrations of cordycepin for 15 min before exposing to Aβ_1–42_ for an additional 24 h. Additionally, the cells were pretreated with PD98059, a specific ERK inhibitor, for 30 min before incubation with cordycepin for 15 min, and thereafter exposure to Aβ_1–42_ for another 24 h. At the end of the experiment, the cell cultures were incubated with a water‐soluble MTT at a concentration of 5 µg/mL for 3–4 h to form the purple formazan. The formazan was subsequently solubilized using DMSO and its concentrations were measured by absorbance at 570 nm using a microplate reader (SpectraMax 250, Molecular Device). The number of living cells was determined by comparison to the percent absorbance of the control group.^34^

#### 2.2.4 ROS staining

The levels of intracellular ROS were measured using the fluorescent probe 2′,7′‐dichlorodihydrofluorescein diacetate (DCFH‐DA) (Beyotime, S0033S), following the protocol provided by the manufacturer. The cell cultures were placed in the dark with DCFH‐DA at a final concentration of 10 μM/L in DMEM without FBS for 30 min. The cells were then washed twice with phosphate‐buffered saline (PBS) solution. Fluorescence was measured using an Infinite M200 PRO Multimode Microplate Reader with a 488 nm excitation wavelength and a 525 nm emission wavelength (TECAN) according to manufacturer protocol.

#### 2.2.5 JC‐1 staining

The mitochondrial membrane potential was determined using the mitochondrial membrane potential assay kit with JC‐1 (Beyotime, C2006), following the protocol provided by the manufacturer. After the appropriate treatment, PC12 cells were incubated with JC‐1 dye at a concentration of 10 μg/mL in a medium without FBS at 37°C for 30 min. Subsequently, the cells were washed two times with a PBS solution. Fluorescence intensities were detected using an M200 PRO Multimode Microplate Reader at red fluorescence with an excitation wavelength of 560 nm and an emission wavelength of 595 nm and green fluorescence with an excitation wavelength of 485 nm and an emission wavelength of 535 nm. The ratio of JC‐1 red/green fluorescence intensity was used to calculate the mitochondrial membrane potential. All values were normalized to the control group.^35^

#### 2.2.6 Flow cytometry

Apoptosis was measured using the flow cytometry assay. Annexin V‐FITC/PI apoptosis detection kit was obtained from BD Biosciences. Briefly, after appropriate treatment, the cells were detached using Trypsin‐EDTA (0.25%) (Thermo Fisher Scientific, 25200056) and suspended in Annexin V‐FITC/PI binding buffer (195 μL). Annexin V‐FITC (5 μL) was added, and the cells were stored in the dark at room temperature for 10–15 min. After centrifugation at 1000 rpm for 5 min, the cells were resuspended in Annexin V‐FITC/PI binding buffer (190 μL). PI (10 μL) was then added, followed by incubation in the dark for 5 min. The apoptotic cells were quantified using BD Accuri™ C6 Plus Flow Cytometer System (C6, BD Bioscience).^36^

#### 2.2.7 CRISPR cas9

CRISPR/Cas9‐mediated gene editing was performed as previously described.^37^ The single guide RNA (sgRNA) targeting ERK1/2 was obtained from Mouse_GeCKOv2_Library_A with the following sequences:

sgRNA (ERK2) oligo1: CACCGTGGCAGAGATGCTATCCAAC and sgRNA (ERK2) oligo2: AAACGTTGGATAGCATCTCTGCCAC; sgRNA (ERK1) oligo1 CACCGCCACTCTGGTCTTGCGCACG and sgRNA (ERK1) oligo2 AAACCGTGCGCAAGACCAGAGTGGC. The sgRNA oligo primers were synthesized by The Beijing Genomics Institute (BGI). Subsequently, the sgRNA was cloned into the pSpCas9(BB)‐2A‐Puro (PX459) V2.0 vector obtained from Addgene (plasmid #62988, Addgene). Verification of the insert's presence involved isolating plasmid DNA from several bacterial colonies and performing sequencing from the U6 promoter (human) (CCGTAACTTGAAAGTATTTCG). The isolated plasmid from positive colonies was then transfected into HT22 cells using Lipofectamine™ 3000 transfection reagent according to the manufacturer's instructions (Thermo Fisher Scientific, L3000015). Cells were selected using 2.5 μg/mL puromycin and cultured in single colonies in 96‐well plates. ERK1/2 expression in both WT and KO cells was assessed by western blot analysis analysis.

#### 2.2.8 Western blot analysis

Western blot analysis was performed according to the previously described procedure.^38^ All cell samples were lysed in ice‐cold radioimmunoprecipitation assay lysis buffer with phenylmethylsulfonyl fluoride (100 mM) (Byotime, T506) and protease‐phosphatase inhibitors cocktail (Universal, 50X) (Beyotime, P1046). The protein concentration was measured using the bicinchoninic acid protein assay kit (absin, abs9232), following the manufacturer's instructions. Equal concentrations of proteins were loaded onto 10% polyacrylamide gels, running for 30 min at 80 V and 90 min at 120 V, and thereafter transferred to a 0.45 μm polyvinylidene fluoride membrane. After blocking with 5% bovine serum albumin for 2 h at room temperature, on a shaker at 100 rpm, the membranes were incubated with specific primary antibodies at 4°C overnight. The following day, primary antibodies were washed three times with 1× TBST for 5 min each time and then incubated with secondary antibodies for an additional 2 h. Subsequently, the membranes were exposed to Pierce™ ECL western blot analysis substrate (Thermo Fisher Scientific, 32209), and the band intensity was quantitated using Image J software. All antibodies are listed in Table 2.

**Table 2:** Detail information of antibodies.

Antibodies	Cat. No	Company	Species	Dilution ratio
Bcl‐2 (D17C4) rabbit mAb	3498s	CST	Rabbit	1:1000
Phospho‐p44/42 MAPK (Erk1/2)(Thr202/Tyr204) antibody	9101	CST	Rabbit	1:1000
ERK 1/2 polyclonal antibody	40902	SAB	Rabbit	1:1000
CREB (86B10) mouse mAb	9104S	CST	Mouse	1:1000
Phospho‐CREB (Ser133) (87G3) rabbit mAb	9198	CST	Rabbit	1:1000
p44/42 MAPK (Erk1/2) (L34F12) mouse	4696s	CST	Mouse	1:1000
Bax antibody	2772s	CST	Rabbit	1:1000
Cleaved caspase‐3	9661s	CST	Rabbit	1:1000
GAPDH	21612	CST	Rabbit	1:1000
Anti‐rabbit IgG, HRP‐linked antibody	7074	CST	Rabbit	1:5000
Anti‐mouse IgG, HRP‐linked antibody	7076	CST	Mouse	1:5000

Abbreviations: CREB, cyclic AMP‐responsive element‐binding protein; CST, Cell Signaling Technology; ERK, extracellular signal‐regulated kinase; GAPDH, glyceraldehyde‐3‐phosphate dehydrogenase; IgG, immunoglobulin G, mAb, monoclonal antibody; MAPK, mitogen‐activated protein kinase

#### 2.2.9 Statistical analysis

Analysis of all data was performed using GraphPad Prism 8 software. Each experiment was performed in triplicates, and all data are expressed as the mean ± standard deviation. Statistical analysis was performed using one‐way analysis of variance followed by Tukey's post hoc test for comparison, considering *p* < 0.05 as statistically significant.

## 3 RESULTS

### 3.1 Cordycepin enhanced cell viability of PC12 cell cultures exposed to Aβ_1–42_ insult

**Figure 1:**
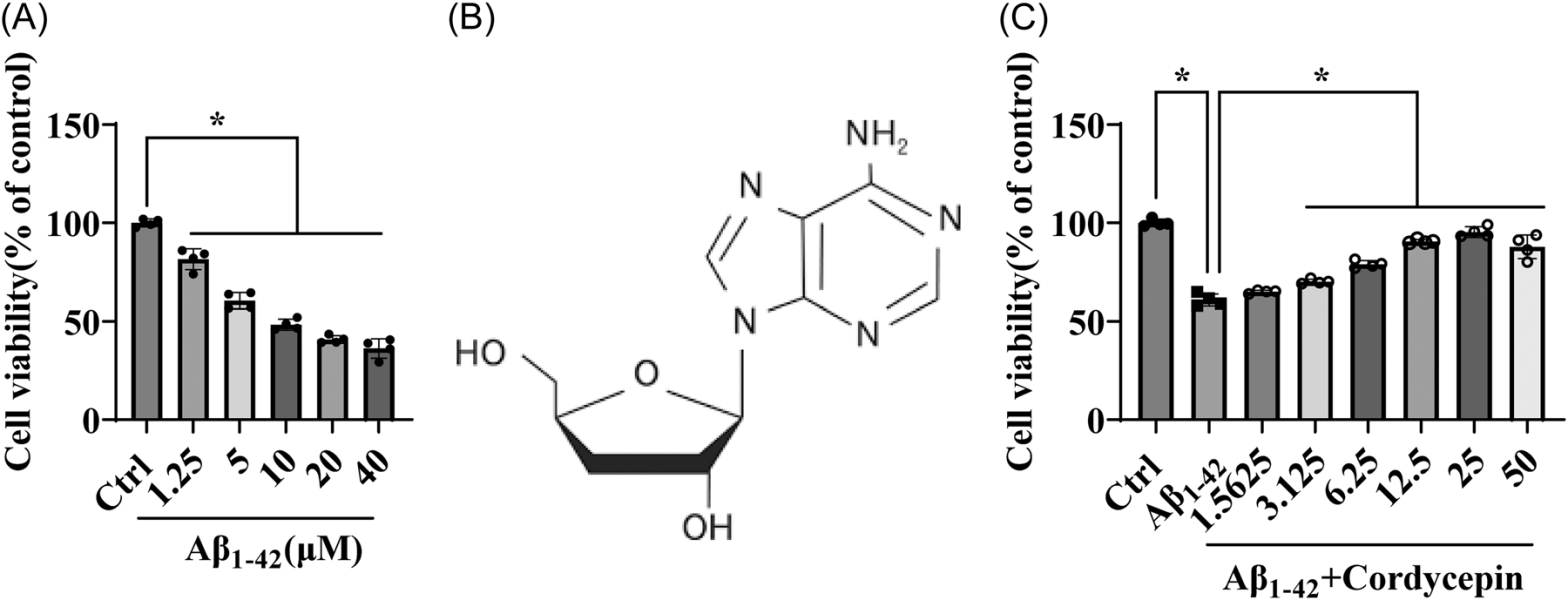
Cordycepin increases the viability of PC12 cells impaired by Aβ_1–42_. (A) Assessment of the neurotoxic effects of Aβ_1–42_. PC12 cells were incubated with different concentrations of Aβ_1–42_ (1.25–40 μM) for 24 h, and cell viability was detected by the MTT assay. (B) Chemical structure of cordycepin. (C) Assessment of the neuroprotective effects of cordycepin treatment on Aβ_1–42_‐induced neurotoxicity. PC12 cells were pretreated with different concentrations of cordycepin for 15 min, followed by incubation with 10 µM Aβ_1–42_ for an additional 24 h. Cell viability was determined by the MTT assay. The experiment was repeated four times, and the data are presented as mean ± SD (*n* = 4). One‐way ANOVA followed by Tukey's post hoc test was used for statistical analysis. *p < 0.05 was considered statistically significant. *Represents a comparison between Ctrl and Aβ_1–42_ and Aβ_1–42_ and cordycepin treatment; Ctrl represents the control group. ANOVA represents analysis of variance; MTT represents 3‐(4,5‐dimethylthiazol‐2‐yl)‐2,5‐diphenyltetrazolium bromide.

To assess the neurotoxic effect of the amyloid peptide in vitro, PC12 cell cultures were treated with Aβ_1–42_ at different concentrations for 24 h. The MTT assay was performed to evaluate the viability of cells, and the results demonstrated that Aβ_1–42_ significantly induced cytotoxicity in a concentration‐dependent manner. This effect was observed starting at a concentration of 10 μM, causing approximately 50% cell death in PC12 cells (Figure 1A). To further investigate the potential protective effect of cordycepin (Figure 1B) against Aβ_1–42_‐induced neurotoxicity, PC12 cells were pretreated with different concentrations of cordycepin for 15 min, before exposure to 10 μM Aβ_1–42_ for an additional 24 h. The results revealed that treatment with cordycepin significantly increased the viability of PC12 cells in a dose‐dependent manner, with the most pronounced effect observed at a dose of 25 μM (Figure 1C). These findings suggest that cordycepin confers neuroprotection toward Aβ_1–42_‐induced neurotoxicity.

### 3.2 Cordycepin reduced apoptosis induced by Aβ_1–42_ in PC12 cell cultures

**Figure 2:**
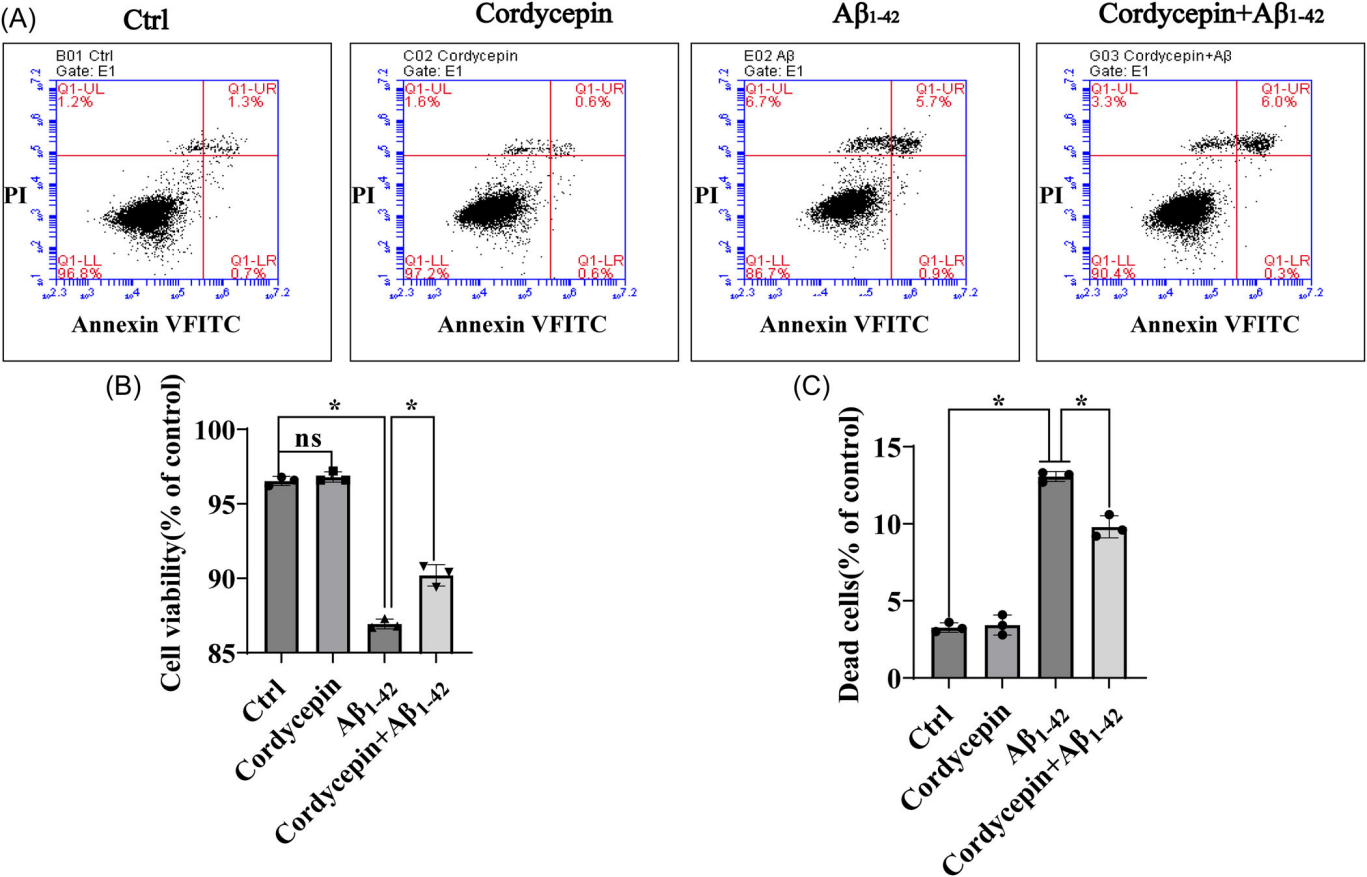
Cordycepin alleviates cellular death induced by Aβ_1–42_ in PC12 cells. PC12 cells were pretreated with 25 µM cordycepin for 15 min and then incubated with or without 10 µM Aβ_1–42_ for another 24 h. (A) Cell apoptosis was analyzed by flow cytometry. (B, C) Quantitative analysis of (A). This assay was performed in triplicate, and the data were presented as mean ± SD (*n* = 3). One‐way ANOVA followed by Tukey's post hoc test was used for statistical analysis. *p < 0.05 was considered statistically significant; ns represents not significant. *Represents a comparison between Ctrl and Aβ_1–42_ and Aβ_1–42_ and cordycepin treatment; Ctrl represents the control group. ANOVA represents analysis of variance. [Color figure can be viewed at wileyonlinelibrary.com]

In another approach, the neuroprotective effect of cordycepin was investigated toward Aβ_1–42_‐induced apoptosis of PC12 cell cultures. Flow cytometry was used to further validate these results. PC12 cells exposed to Aβ_1–42_ insult displayed apoptotic cell death, and 25 μM cordycepin significantly decreased by approximately 4% the apoptosis (Figure 2A–C). Cumulatively, these experiments indicate that cordycepin conferred neuroprotection to PC12 cell cultures exposed to Aβ_1–42_‐insult.

### 3.3 Cordycepin alleviated intracellular ROS levels and restored mitochondrial membrane potential in PC12 cell cultures exposed to Aβ_1–42_ insult

**Figure 3:**
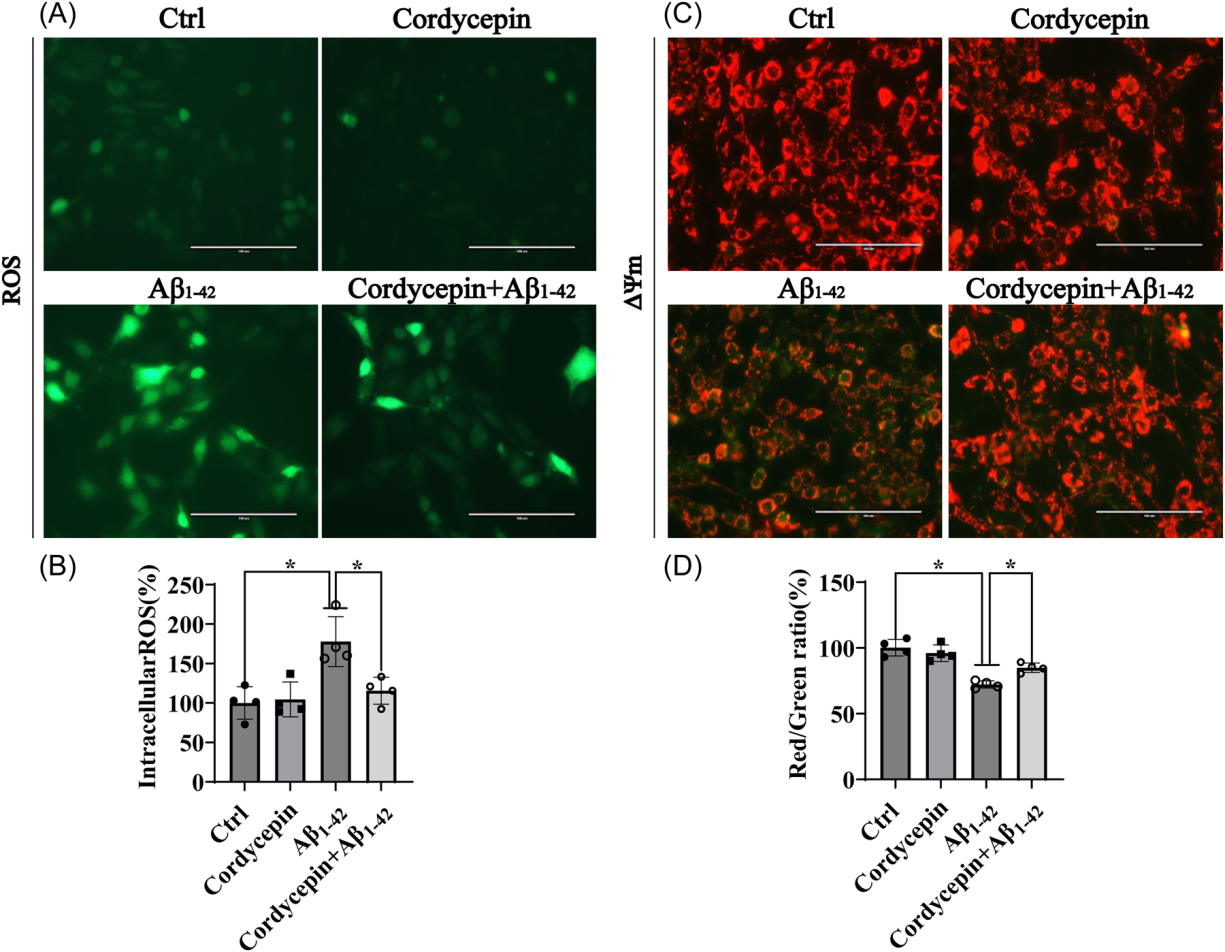
Cordycepin reduces intracellular ROS levels and restores mitochondrial membrane potential in Aβ_1–42_‐induced PC12 cell model. PC12 cells were pretreated with 25 µM cordycepin for 15 min and then incubated with or without 10 µM Aβ_1–42_ for an additional 24 h. (A, B) Intracellular ROS levels were measured by ROS assay, and statistical analysis indicated that cordycepin inhibited the increase in intracellular ROS levels. (C, D) Mitochondrial membrane potential was assessed by JC‐1 assay, and statistical analysis demonstrated that cordycepin promoted the recovery of mitochondrial membrane potential. This experiment was performed in triplicate (*n* = 4, mean ± SD). One‐way ANOVA followed by Tukey's post hoc test was used for statistical analysis. *p < 0.05 was considered statistically significant, ns represents not significant. *Represents a comparison between Ctrl and Aβ_1–42_; Aβ_1–42_ and cordycepin treatment; Ctrl represents the control group. ANOVA represents analysis of variance; ROS represents reactive oxygen species; Δψm represents mitochondrial membrane potential. [Color figure can be viewed at wileyonlinelibrary.com]

To explore whether cordycepin reduced the increase of intracellular ROS and restored the effect on mitochondrial membrane potential, PC12 cell cultures were incubated with 25 μM cordycepin for 15 min and then exposed to 10 μM Aβ_1–42_ for another 24 h. The results indicated that the increase of intracellular ROS of about 70% over the control caused by Aβ_1–42_ was significantly abolished in PC12 cell cultures treated with cordycepin (Figure 3A,B). The loss of mitochondrial membrane potential induced by Aβ_1–42_ was reversed after cordycepin treatment (Figure 3C,D). These findings point to the ability of cordycepin to protect mitochondria from Aβ_1–42_‐induced mitochondrial dysfunction and intracellular ROS.

### 3.4 Cordycepin increased the phosphorylation levels of ERK and CREB

**Figure 4:**
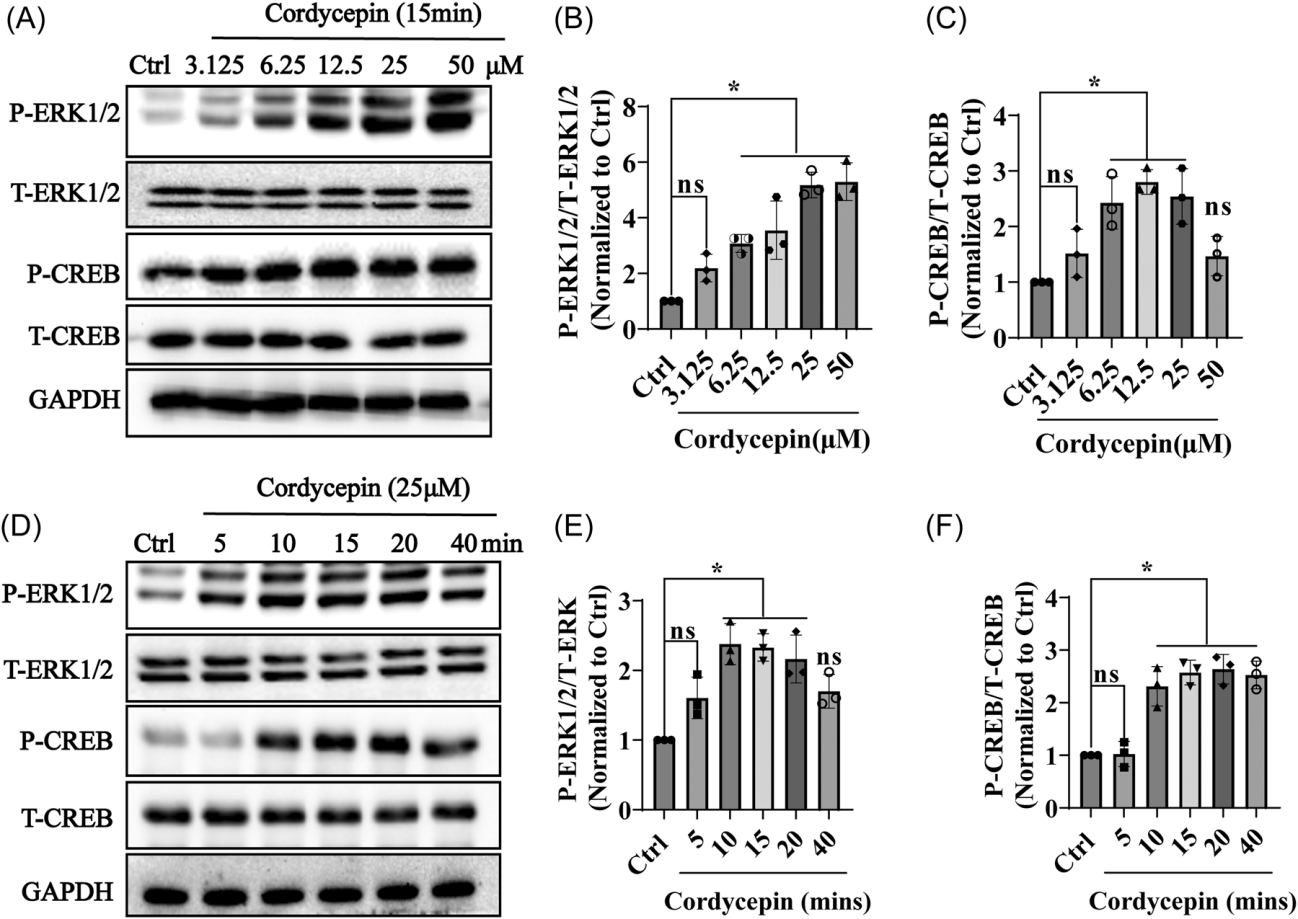
Cordycepin stimulates phosphorylation of ERK and CREB in PC12 cells. (A) PC12 cells were collected after treatment with different concentrations of cordycepin (0, 3.15, 6.25, 12.5, 25, and 50 µM) for 15 min and then cellular proteins were collected and analyzed for the expression levels of P‐ERK1/2, T‐ERK1/2, P‐CREB, T‐CREB, and GAPDH through western blot analysis. (B, C) Quantitative analysis of (A). (D) PC12 cells were incubated with 25 µM cordycepin for different times (0, 5, 10, 15, 20, 40 min), and then cellular proteins were collected and analyzed for the expression levels of P‐ERK1/2, T‐ERK1/2, P‐CREB, T‐CREB, and GAPDH through western blot analysis. (E, F) Quantitative analysis of D. The assay was repeated three times (*n* = 3, mean ± SD). One‐way ANOVA followed by Tukey's post hoc test was used for statistical analysis. *p < 0.05 was considered statistically significant; ns was considered not statistically significant. *Represents a comparison between Ctrl and cordycepin treatment; Ctrl represents the control group. ANOVA, analysis of variance; CREB, cyclic AMP‐responsive element‐binding protein; ERK, extracellular signal‐regulated kinase; GAPDH, glyceraldehyde‐3‐phosphate dehydrogenase; P‐CREB, phosphorylated‐CREB; P‐ERK1/2, phosphorylated‐ERK1/2; T‐CREB, Total‐CREB; T‐ERK1/2, Total‐ERK1/2.

To investigate whether ERK‐CREB is involved in the neuroprotective effects of cordycepin, PC12 cell cultures were incubated for 15 min with different concentrations of cordycepin, and the phosphorylation levels of ERK1/2 and CREB were measured by western blot analysis. Cordycepin dose‐dependently increased the phosphorylation of ERK1/2 and CREB (Figure 4A–C). Subsequently, PC12 cells were treated with 25 μM cordycepin from 0 to 40 min and the results demonstrated that treatment with cordycepin for 15 min significantly promoted the phosphorylation of ERK1/2 and CREB (Figure 4D–F). These data indicate that cordycepin stimulated the phosphorylation of ERK and CREB.

### 3.5 ERK/CREB pathway is involved in cordycepin‐induced neuroprotection of PC12 cell cultures exposed to Aβ_1–42_ insult

**Figure 5:**
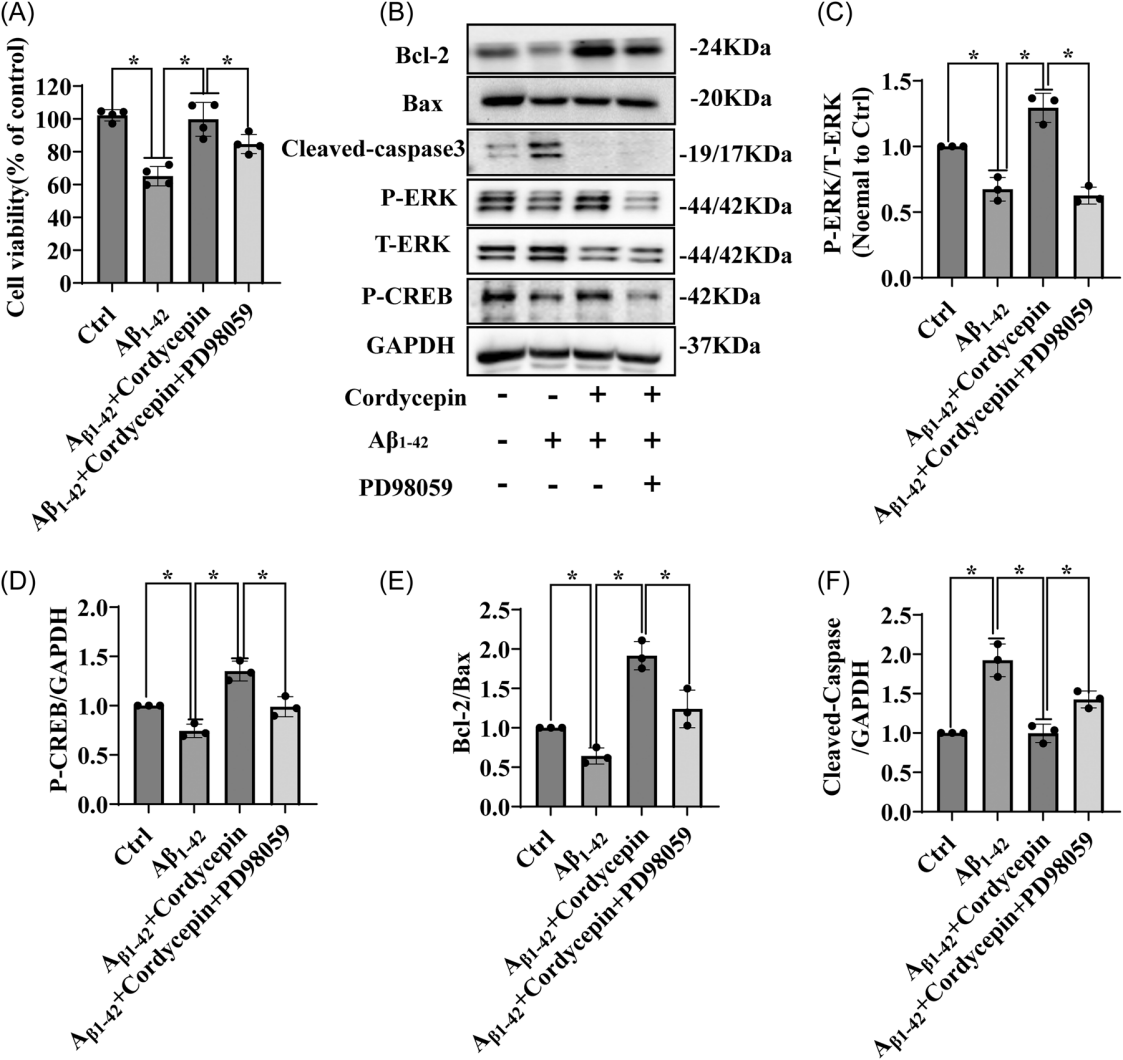
ERK/CREB pathway mediates the protective effect of cordycepin in PC12 cells. (A) PC12 cells were pretreated with 25 µM PD98059 for 30 min, followed by treatment with 25 µM cordycepin for 15 min. Subsequently, the cells were incubated with or without 10 µM Aβ_1–42_ for an additional 24 h, and cell viability was measured by the MTT assay. The experiment was repeated four times (*n* = 4, mean ± SD), *p < 0.05 was considered statistically significant, *Represents a comparison between Ctrl and Aβ_1–42_ group; Aβ_1–42_ and treatment group; treatment group and inhibitor group; Ctrl represents the control group. (B) Western blot analysis was performed to assess the expression of P‐ERK, T‐ERK, P‐CREB, T‐CREB, cleaved‐caspase 3, Bcl‐2, Bax, and GAPDH. (C–F) Quantitative analysis of the results presented in B. The assay was performed in triplicate, and the experiment was repeated three times (*n* = 3, mean ± SD). One‐way ANOVA followed by Tukey's post‐hoc test was used for statistical analysis. *p < 0.05 was considered statistically significant; ns was considered not statistically significant. *Represents a comparison between Ctrl and Aβ_1–42_ group; Aβ_1–42_ and treatment group; treatment group and inhibitor group. Ctrl represents the control group. ANOVA, analysis of variance; Bax, Bcl‐2‐like protein 4; Bcl‐2, Bcl‐2 apoptosis regulator; CREB, cyclic AMP‐responsive element‐binding protein; ERK, extracellular signal‐regulated kinase; GAPDH, glyceraldehyde‐3‐phosphate dehydrogenase; P‐CREB, phosphorylated‐CREB; P‐ERK, phosphorylated‐ERK; T‐CREB, Total‐CREB; T‐ERK, Total‐ERK.

Considering previous data indicating that cordycepin stimulates the activation of the ERK‐CREB signaling pathway in PC12 cell cultures, we aimed to investigate whether this pathway is also involved in the neuroprotective effect of cordycepin against cellular apoptosis induced by Aβ_1–42_. To this goal, PC12 cell cultures were pretreated for 30 min with 25 μM PD98059 (a specific ERK inhibitor) and subsequently treated with cordycepin for 15 min before being exposed to Aβ_1–42_ for an additional 24 h. Cell viability was assessed using the MTT assay, and the results showed that PD98059 blocked the neuroprotection induced by cordycepin (Figure 5A). Western blot analysis further revealed that, although cordycepin treatment reversed Aβ_1–42_‐induced the decrease in phosphorylation of ERK and its downstream target CREB, this effect was suppressed with the inhibition of ERK by PD98059, indicating that the ERK‐CREB axis plays a crucial role in the protective effect of cordycepin (Figure 5B–D). Moreover, the cordycepin‐induced increase in the Aβ_1–42_‐induced decrease of the Bcl‐2/Bax expression ratio was prevented by PD98059 (Figure 5B,E). Additionally, the increase in cleaved‐caspase 3 expression induced by Aβ_1–42_ was significantly attenuated after treatment, an effect that was blocked by PD98059 (Figure 5B,F). These findings demonstrate that ERK‐CREB signaling is involved in the neuroprotective effects of cordycepin against Aβ_1–42_‐induced insult.

### 3.6 ERK/CREB pathway is involved in cordycepin‐induced neuroprotection of hippocampal neurons exposed to Aβ_1–42_ insult

**Figure 6:**
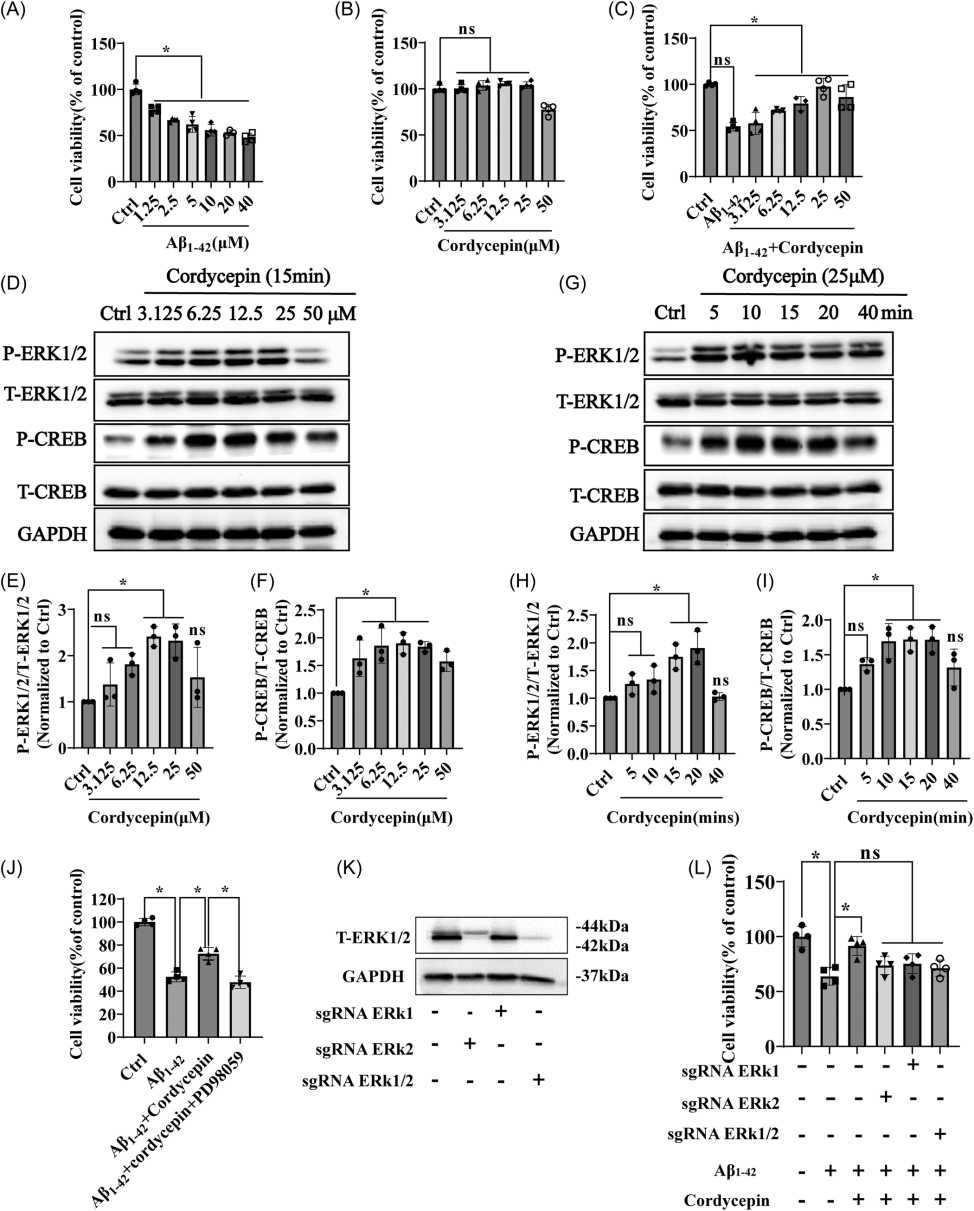
ERK/CREB signaling mediates the protective effect of cordycepin in HT22 cells. (A) Assessment of cytotoxicity of Aβ_1–42_ on HT22 cells. (B) Measurement of cordycepin concentration. (C) HT22 cells were pretreated with cordycepin at indicated concentrations and then induced with or without 10 µM Aβ_1–42_ for an additional 24 h; cell viability was measured using the MTT assay. The experiment was repeated four times (*n* = 4, mean ± SD, *p < 0.05 was considered statistically significant); Ctrl represents the control group. (D) Western blot analysis of P‐ERK, T‐ERK, P‐CREB, and GAPDH. Cordycepin increased the phosphorylation levels of P‐ERK and P‐CREB in a dose‐dependent manner. (E, F) Quantitative analysis of the results presented in D. (G) Western blot analysis of P‐ERK, T‐ERK, P‐CREB, and GAPDH in a time‐dependent manner. (H‐I) Quantitative analysis revealed that cordycepin increased the phosphorylation levels of P‐ERK and P‐CREB in a time‐dependent manner. This experiment was repeated three times (*n* = 3, mean ± SD). (J) HT22 cells were pretreated with 25 µM PD98059 for 30 min, treated with 25 µM cordycepin for 15 min, followed by incubation with 10 µM Aβ_1–42_ for another 24 h. Cell viability was assessed by the MTT assay. (K) Expression of total ERK and GAPDH measured by Western blot. (L) HT22 cells with or without knockout were incubated with 25 µM cordycepin for 15 min, exposed to 10 µM Aβ_1–42_ for an additional 24 h, and cell viability was measured by the MTT assay. The assay was repeated four times (*n* = 4, mean ± SD). *p < 0.05 was considered statistically significant. *Represents a comparison between Ctrl and Aβ_1–42_ group; Aβ_1–42_ and treatment group; Ctrl represents the control group. CREB, cyclic AMP‐responsive element‐binding protein; ERK, extracellular signal‐regulated kinase; GAPDH, glyceraldehyde‐3‐phosphate dehydrogenase; P‐CREB, phosphorylated‐CREB; P‐ERK1/2, phosphorylated‐ERK1/2; T‐CREB, Total‐CREB; T‐ERK1/2, Total‐ERK1/2.

To further confirm that ERK/CREB pathway is involved in cordycepin‐induced neuroprotection, we investigated HT22 hippocampal neurons exposed to Aβ_1–42_ insult. The findings indicate that cordycepin effectively protected hippocampal neurons against Aβ_1–42_‐induced neurotoxicity of HT22 cells (Figure 6A–C). Moreover, HT22 cells were exposed to different concentrations of cordycepin for 15 min, and protein samples were evaluated by western blot analysis indicating a dose‐dependent increase in the phosphorylation of ERK1/2 and CREB (Figure 6D–F). HT22 cells were also treated with a single concentration of 25 µM cordycepin for different periods of time and a time‐dependent increase in the phosphorylation levels of ERK1/2 and CREB was observed (Figure 6G–I). These results provide an apparent temporal link between cordycepin‐induced neuroprotection of HT22 cell cultures exposed to Aβ_1–42_ insult and the activation of the ERK/CREB signaling pathway. To further explore the involvement of the ERK/CREB signaling pathway in the neuroprotective effect of cordycepin, the cell cultures were incubated with 25 µM PD98059 for 30 min. Subsequently, the cells were treated with 25 µM cordycepin for 15 min before exposure to 10 µM Aβ_1–42_ for an additional 24 h. The cell viability was assessed using the MTT assay. The results indicated that treatment with PD98059 blocked the neuroprotective effect of cordycepin (Figure 6J). In another approach, the simultaneous knockout of both ERK1/2 was performed using CRISPR Cas9 technology. The quality of cell cultures with the double knockouts was confirmed by western blot analysis (Figure 6K). Thereafter, the HT22 cell cultures were pretreated with cordycepin for 15 min before exposure to Aβ_1–42_ for an additional 24 h. MTT assay results demonstrated that blocking the ERK/CREB signaling pathway by knocking out ERK1 and ERK2, respectively, inhibited the neuroprotective effect of cordycepin (Figure 6L). These findings collectively suggest that the ERK/CREB signaling pathway is involved in the neuroprotection of cordycepin toward Aβ_1–42_‐induced cellular apoptosis.

## 4 DISCUSSION

In this study, cellular models of PC12 and HT22 neuronal cell cultures were investigated to prove the concept that cordycepin‐induced in vitro neuroprotection toward Aβ_1–42_ neurotoxic insult. The results indicate that acute short treatment with cordycepin conferred neuroprotection to PC12 and HT22 neuronal cell cultures exposed to Aβ_1–42_‐induced apoptotic cell death. Consistent with others^39^ and our previous reports,^31^ we observed that Aβ_1–42_ is neurotoxic to PC12 cells in a dose‐dependent manner. In addition, results obtained from flow cytometry and western blot analysis suggested that cordycepin decreased cell apoptosis caused by Aβ_1–42_, and the MTT assay also showed a significant increase in cell viability. Cordycepin alleviated intracellular ROS levels and restored mitochondrial membrane potential in PC12 cell cultures exposed to Aβ_1–42_ insult. These findings are in line with previous studies indicating that cordycepin inhibited 6‐hydroxydopamine‐induced apoptosis and mitochondrial dysfunction in PC12 cell cultures by its potent antioxidant activity,^40^ alleviated oxidative stress in neuroblastoma cell cultures,^41^ and conferred antiapoptotic effects in hippocampal primary neurons^42^ and in hippocampal HT22 cell cultures exposed to glutamate‐induced oxidative stress.^43^

Based on the cell death experiments and measurements of the phosphorylation of ERK/CREB proteins in neuronal cells exposed to Aβ_1–42_ insult in the presence or absence of an ERK inhibitor, and in cell cultures knockout for ERK1/2 proteins, a clear apparent temporal link between cordycepin‐induced neuroprotection and the activation of the ERK/CREB signaling pathway was found. The ERK signaling pathway participates in the process of neuronal apoptosis. Although the majority of studies demonstrate an antiapoptotic role in neurons as found here, a dual role in the apoptosis of neurons has also been reported.^44^ Compelling evidence proposed that ERK activation may be related to either neuronal cell death or survival depending on the insult type, intensity, duration, the type of its cellular substrates that are phosphorylated, and its cellular compartmentalization.^45^ ERK pathway is a canonical prosurvival signaling pathway in neurons that is activated by neurotrophins.^46^ ERK pathway is critical in mediating protection against apoptotic cell death induced by either trophic factor withdrawal or increased oxidative stress.^47^ It promotes cell survival not only by inhibiting the expression of apoptotic genes but also by blocking the activity of proapoptotic proteins in mitochondria such as caspases. In addition, ERK cassette signaling also regulates intracellular oxidation state through various pathways, such as the Nrf2‐Keap1 pathway.^48^ Thus, activation of ERK phosphorylation by cordycepin can explain its neuroprotective effect in neuronal cultures toward Aβ_1–42_‐induced pathological processes such as increase of oxidative stress, loss of mitochondria membrane potential, and activation of caspase 3. CREB is an important transcription factor that regulates neuronal growth, neuronal differentiation and proliferation, synaptic plasticity, neurogenesis, maturation of neurons, and memory, as well as ensures neuronal survival in AD.^49^ Several kinases, including ERK, phosphorylate CREB and affect its transcriptional activation. Several studies have also shown that CREB activation mitigates apoptosis mediated by oxidative stress.^50^, ^51^ ERK/CREB signaling is linked to brain‐derived neurotrophic factor which is involved in the regulation of cognitive functions and neuronal plasticity and contributes to the management of AD.^52^ For this reason, many drugs^48^ and traditional Chinese medicines^53^ are targeting CREB for the treatment of oxidative stress conditions in AD. Thus, activation of CREB phosphorylation by cordycepin may explain its neuroprotective effect on neuronal cultures exposed to Aβ_1–42_ apoptotic insult. Albeit these in vitro experiments are the prerequisite for future drug development, further characterization of the neuroprotective effect of cordycepin is also required in pharmacological preclinical in vivo models such as the triple transgenic AD,^54^ reflecting the plaque and tangle pathology association with the synaptic dysfunction.^55^

Multiple studies have shown that activation of the ERK/CREB pathway plays a key role in neuroprotection.^34^, ^36^ Present findings using in vitro neuronal cultures propose cordycepin as a natural lead compound for therapeutic interventions targeting Aβ_1–42_‐induced neurotoxicity in AD. The implication of the ERK/CREB signaling pathway in the neuroprotective effects of cordycepin provides valuable insight for future drug development efforts aiming to harness the neuroprotective potential of cordycepin.

## 5 CONCLUSION

In conclusion, cordycepin could effectively protect PC12 and HT22 neuronal cell cultures from Aβ_1–42_‐induced apoptosis. Cordycepin significantly reduced the production of ROS, restored mitochondrial membrane potential, and inhibited apoptosis. Cordycepin‐induced activation of the ERK/CREB signaling pathway in temporal correlation with the neuroprotective effect. Further exploration of the neuroprotective function of cordycepin and related signaling pathways could pave the way for the development of innovative therapies for AD and related neurodegenerative disorders.

## AUTHOR CONTRIBUTIONS

Wenshu Zhou performed the experiments and drafted the manuscript. Cheng Wang and Yige Tan performed part of the experiments. Xiaoyan Wen and Philip Lazarovici revised the manuscript. Wenhua Zheng and Shaoping Li conceived the hypothesis, designed the experiments, and revised the manuscript. All authors read and approved the final manuscript.

## CONFLICT OF INTEREST STATEMENT

The authors declare no conflicts of interest.

## ETHICS STATEMENT

The cell lines used in this study, PC12 cells and HT22 cells, were provided by Yat‐sen University's Cell Bank. The study does not involve any animal experiments and does not raise any ethical concerns.

## Data Availability

All data in this study are available from the corresponding author upon reasonable request.
